# The severity of LPS induced inflammatory injury is negatively associated with the functional liver mass after LPS injection in rat model

**DOI:** 10.1186/s12950-018-0197-4

**Published:** 2018-11-15

**Authors:** Haoshu Fang, Anding Liu, Xulin Chen, Wenhui Cheng, Olaf Dirsch, Uta Dahmen

**Affiliations:** 1 Department of Pathophysiology, Anhui Medical University, Hefei, 230032 China; 2 Experimental Transplantation Surgery, Department of General, Visceral and Vascular Surgery, Friedrich-Schiller-University Jena, Drackendorferstraße1, 07747 Jena, Germany; 3 Laboratory Animal Research Center, College of Basic Medical Sciences, Anhui Medical University, Hefei, China; 4 Experimental Medicine Center, Tongji Hospital, Tongji Medical College, Huazhong University of Science and Technology, Wuhan, 430030 China; 5 Department of Burns, the First Affiliated Hospital of Anhui Medical University, Hefei, 230022 China; 6 Institute of Pathology Hospital of Chemnitz, Chemnitz, Germany

**Keywords:** LPS, Hepatic LPS uptake, SIRS

## Abstract

**Background:**

High levels of serum lipopolysaccharide (LPS) were observed in sepsis patients with liver injury and high mortality. However, the role of liver in modulation LPS induced inflammatory injury was ill investigated. In the present study, the severity of LPS induced inflammatory response was observed after liver resection or portal branch occlusion to decreasing functional liver mass. The local and systemic damage was observed to investigate the role of liver in modulation inflammatory injury.

**Methods:**

First, 30%, 70%, and 90% partial hepatectomy (PH) were performed, and serum TNF-α, survival rate, and hepatic LPS uptake was observed. Second, LPS-exposure of the functional liver mass was decreased by selectively blocking the RL prior to LPS-injection, which was given 30 min before a 70% PH, and the inflammatory response was compared in the occluded and the non-occluded liver. The control group was subjected to LPS injection 30 min prior to liver resection without blocking the RL transiently. The serum TNF-α, ALT, AST, creatinine levels, and urea levels, survival rate, hepatic LPS uptake, and hepatic inflammatory cytokines was observed.

**Results:**

The decreasing of functional liver mass after 90%, 70%, and 30% PH was associated with decreased serum TNF-α, survival rate, and increased hepatic LPS uptake after LPS injection. Occluding the right lobes (RL) prior to LPS administration reversed the liver injury caused by 70% PH, indicated by 100% survival rate and decreased liver and kidney injury, and systemic inflammatory response. The induction of inflammatory response in occluding liver lobes were lower than un-occluding liver lobes.

**Conclusions:**

The severity of the LPS-induced systemic inflammatory injury is determined by functional liver volume. This observation suggests that the liver is the central organ for the initiation of the inflammatory response, and is involved in causing a severe SIRS with systemic damage and death.

**Electronic supplementary material:**

The online version of this article (10.1186/s12950-018-0197-4) contains supplementary material, which is available to authorized users.

## Introduction

SIRS leads to multi-organ dysfunction (MOD), such as the hepato-renal and hepatopulmonary syndrome. Initiation and course of MOD is not well understood. It is not known which organ primarily triggers the LPS-response and induces the damage and functional impairment in other organs. The standard model of SIRS is the endotoxemia model. Typically, endotoxemia is induced by intraperitoneal or intravenous injection of LPS. In these models, the inflammatory injury can be observed on a systemic level.

LPS is the main component of gram negative bacteria, and induces an inflammatory response in mammalian species [1]. LPS binds to LPS binding protein (LBP), and CD14, which initiates the inflammatory response by triggering the production of inflammatory cytokines via activation of the TLR4 signal pathway [2].

The activation of inflammatory response is beneficial for inducing LPS and bacterial clearance. However, the inflammatory response may become deleterious if circulatory LPS become abundant, leading to tissue damage, multi-organ dysfunction, and death [3].

The contributions of the individual organ such as the liver to the systemic inflammatory response remain unclear. The pathophysiological relationship between endotoxemia, SIRS, and multi-organ dysfunction is not well explored, in part because of the lack of suitable animal models.

In the present study, we established a new model to investigate the role of liver in triggering and modulating the LPS induced systemic response and injury. In this model, the hepatic LPS uptake was increased by either removing or occluding liver lobes. Our results indicated that decreasing hepatic LPS uptake by transient occlusion of the right liver lobes of a rat decreased the local hepatic as well as the systemic injury, and the over-all mortality.

## Materials and methods

### Experimental design

The relationship between liver mass and induction of inflammatory response was explored by performing partial hepatectomy (PH) removing 30%, 70%, and 90% of the liver mass immediately before LPS administration (2 mg/kg, intravenous injection, *E. coli* serotype O55:B05 type, Sigma-Aldrich, St. Louis, USA). Survival rate at 24 h was observed (*n* = 6 per group). In order to investigate the induction of inflammatory response, rats were sacrificed 1 h after LPS injection (*n* = 3 per group), and the histology, serum TNF-α levels, and hepatic LPS uptake were used as read-out parameter.

In order to investigate the effect of liver volume and LPS exposure on the severity of inflammatory injury, a novel surgical model was designed. The hepatic LPS exposure was inhibited by occlusion of the right liver lobes immediately before LPS injection. The 70% PH was performed 30 min after LPS injection (Additional file 1: Figure S1). In control group, the LPS was injected, and the 70% liver mass was recected without RL occlusion. Survival rate was determined within a 24 h observation period (*n* = 6). In order to investigate the effect of hepatic LPS exposure on inflammatory severity, a kinetic experiment was performed. The LPS (2 mg/kg) was injected, and the rats were sacrificed at 0.5 h, 1 h, 6 h, and 24 h (*n* = 6 per group). Blood samples as well tissue samples from liver, and kidney were taken for further investigation. Induction and severity of the inflammatory response in terms of survival rate, systemic and local inflammatory injury was compared.

### Animals

Male Lewis rats (12 weeks old, body weight 250 ± 50 g; Charles River, Sulzfeld, Germany) were used in this study. All animals were housed under standard animal care conditions and had free access to water and rat chow ad libitum. Animals were allowed to adapt to laboratory conditions for 7 days. The permission for animal experiments was given by the “Thüringer Landesamt für Verbraucherschutz; AZ: 2226840402026/13), and were performed under inhalation anesthesia with 3% isoflurane (Sigma Delta, London, UK).

### Surgical model

Liver resection was performed by removing the left lateral lobe (30%PH) and the median lobe (about 70% liver volume) and the right lobes (90%PH). Blood samples were taken repeatedly every 15 min until 90 min for assessment of systemic TNF-a level. One group of animals was observed for up to 24 h to determine the survival rate.

In the clamping experiment, the rats were divided to LPS + 70% PH group and liver occlusion+LPS + 70% PH group. In LPS + 70% PH group, the rats received an intravenous LPS-injection (2 mg/kg) immediately after removing of 70% liver mass (median lobe + left lateral lobe). In liver occlusion+LPS + 70%PH group, the blood flow to right lobes was blocked by clamping the portal branch of right lobes (20% liver mass). After right lobes ischemia, LPS (2 mg/kg) was injected intravenously. Then, the right lobes were re-perfused 30 min after LPS injection, and 70% of liver mass (median lobes + left lateral lobe) was resected at the same time (Additional file 1: Figure S1).

### Organ injury

Liver injury was investigated by measuring the serum level of liver enzymes (AST, and ALT) using an Automated Chemical Analyzer (Bayer Advia 1650; Leverkusen, Germany). Kidney damage was assessed by measuring serum creatinine and urea levels using the Automated Chemical Analyzer.

### Histological staining

Liver, kidney tissues were fixed in 4.5% buffered formalin for 48 h, and were embedded by paraffin. Sections of 4 μm thickness were cut and stained with Hematoxylin-Eosin. The stained slides were digitalized using a slides scanner (Hamamatsu Electronic Press Co., Ltd., Lwata, Japan), and evaluated by one experienced pathologist (OD), and two experienced scientists (FH, and LA), according to a standardized semi-quantitative scoring system as described before [4].

### LPS-immunohistochemistry (IHC)

Liver sections were used for LPS staining. The antigen retrieval was performed by using 10 mM Citrate buffer (pH 6.0) for 20 min at room temperature. Sections were washed 3 times with TBST, and incubated with the hydrogen peroxide for 5 min. After another 3 washes with TBST, sections were incubated with protein blocking buffer. After protein blocking, the slides were incubated with polyclonal mouse anti-LPS antibody (1:100, Abcam, Cambridge, UK) for 15 min. Signals were amplified by using CSAII system (Dako, Glostrup, Denmark), and the counterstaining was performed by dipping the slides into Hematoxylin for 5 min. The stained sections were digitalized using the slide scanner, and evaluated as described before [5]. A semi-quantitative system was used to evaluate the LPS staining: negative staining = < 10% positive cells; less positive staining = 10–40% positive cells; moderate positive staining = 40–70% positive cells; positive staining = 70–100% positive cells.

### Naphthol-AS-D-chloroacetate Esterase (ASDCL) staining

Hepatic neutrophil infiltration was evaluated by ASDCL staining as reported previously. The neutrophil infiltration was evaluated by selecting 10 high-power fields (HPF) pictures with the magnification of 200 × randomly, followed by manual counting of all positively staining neutrophils. The results were analyzed by calculating the mean number of ASDCL positive neutrophils per HPF.

### Enzyme-linked Immunosorbent Assay (ELISA)

The commercially available ELISA kits (R&D Systems, Minneapolis, US) were used for detecting serum TNF-α level. The procedure was done according to the manufacturers suggestions. The plates were measured by using an ELx 808 ELISA reader (Bio-Tek Instruments Inc., Winooski, VT, US) at 450 nm.

### Quantitative polymerase chain reaction (PCR)

Total RNA was isolated from liver, and kidney tissues using the RNeasy kit (Qiagen, Hilden, Germany), and the complementary DNA was synthesized using the First-Strand cDNA synthesis kit (Invitrogen, Carlsbad, USA). The quantitative PCR reaction was performed by using 1 μg of cDNA, and Brilliant qPCR Master Mix kit (Agilent, Santa Clara, USA). The sequences of primers and probes were listed: hypoxanthine guanine phosphoribosyltransferase (HPRT): GACCGGTTCTGTCATGTCG, ACCTGGTTCATCATCACTAATCAC, and Probe #95; TNF-α: TGAACTTCGGGGTGATCG, GGGCTTGTCACTCGAGTTTT, and Probe #63; IL-6: CCTGGAGTTTGTGAAGAACAACT, GGAAGTTGGGGTAGGAAGGA, and Probe #106; CCL2: AGCATCCACGTGCTGTCTC, GATCATCTTGCCAGTGAATGAG, and Probe #62; CCL3: GCGCTCTGGAACGAAGTCT, GAATTTGCCGTCCATAGGAG, and Probe #40. The standard curve was generated using a serial dilution of a normal sample. Gene expression was normalized using hypoxanthine guanine phosphoribosyltransferase (HPRT) [6], and the fold change was calculated using a normal liver tissue sample as reference sample.

### Statistical analysis

All values were expressed as mean ± SD. All statistical calculations were performed by using Sigma Stat (ver. 3.5.54; Systat Software GmbH, Erkarth, Germany). Groups of animals were compared employing Student’s t-test in case of normal distribution of the data. If data were not normally distributed, the Mann – Whitney rank sum test was employed to compare sets of data in different animal groups. *P* < 0.05 was considered as statistical significant.

## Results

### The severity of the systemic inflammatory response was related to liver mass

We administrated a sub-lethal dose of LPS (2 mg/kg) immediately after removing either 30%, 70%, or 90% liver mass. Without LPS-administration, all three resection models are survival models [5]. However, in case of an additional otherwise sub-lethal LPS-injection immediately after completing the resection, the survival rate was related to the size of the remnant liver. All rats survived after 30% PH and LPS administration. After 70% PH, 66% of rats did not tolerate the otherwise sub-lethal LPS dose and died with 20 h. In contrast, no rat tolerated the same LPS dose after 90% PH with a survival time below 24 h.

### Inflammatory response: Systemic TNF-a level were associated with the size of the remnant liver mass after hepatectomy followed by LPS injection

**Fig. 1:**
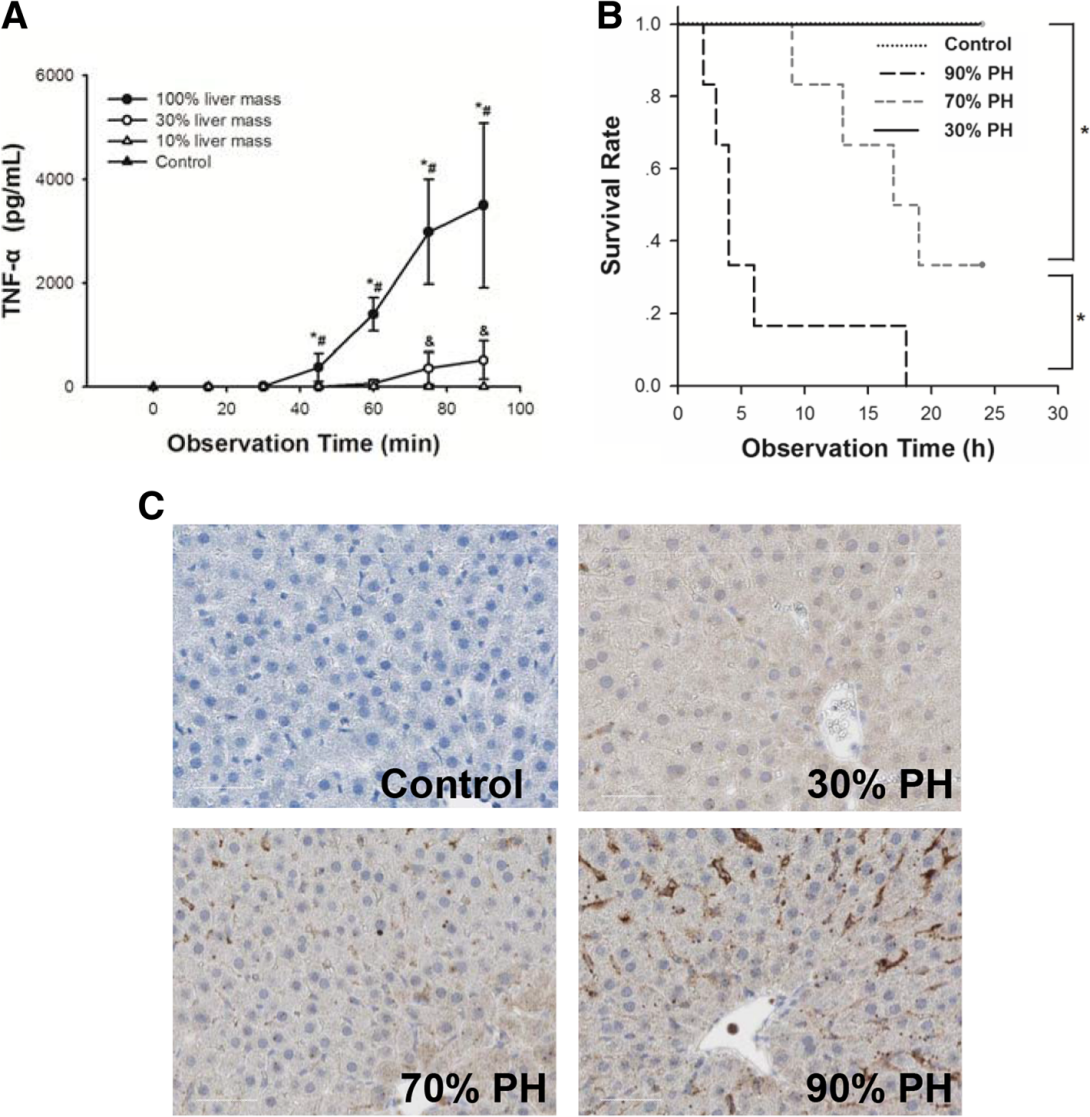
The severity of the systemic inflammatory response was related to the liver mass. **a** The serum TNF-α levels after 30% PH, 70% PH, and 90% PH with LPS injection; **b** the survival rate after 30% PH, 70% PH, and 90% PH with LPS injection, **c** LPS IHC staining was performed to assess the hepatic uptake of LPS. Original magnification × 400. Representative images from 6 rats per group were selected; Data were shown as mean ± SD, *n* = 3 per group; * *p* < 0.05 30% PH group vs.70% PH group, #*p* < 0.05 30% PH group vs. 90% PH group, &*p* < 0.05 70% PH group vs. 90% PH group

The relationship between liver mass and the induction of systemic TNF-α levels were investigated by removal of liver mass. Reduction of liver mass was associated with decreased systemic TNF-α level, but a higher mortality, as outlined above (Fig. 1a-b). When removing 30% of the liver mass, serum TNF-α levels reached more than 3000 pg/ml (3501 pg/ml ± 1587). After 70%PH, the serum TNF-α levels were significantly lower. Of note, after 90% PH, the serum TNF-α was almost undetectable (Fig. 1a). Reduction of the liver mass was associated with lower systemic TNF-α levels; in other words: TNF-α release seemed to be inversely related to the extent of liver resection, suggesting that systemic TNF-α is derived from the liver.

### Hepatic LPS uptake was related to the volume of remnant liver mass

After minor 30% PH (less positive staining) and subsequent sublethal LPS-injection little LPS uptake was observed and slightly more after 70% PH (moderate positive staining). Of note, after 90% PH (positive staining), the hepatic LPS uptake was substantially higher than in both other groups (Fig. 1c). These observations suggest that primary binding of LPS occurs in the liver, since all circulating LPS is somehow accumulating in the small remnant liver.

### Reducing LPS-load of the remnant liver by partial portal vein occlusion reduced the lethal effect of LPS

Based on these observations, we hypothesized, that the severity of LPS induced inflammatory response might be determined by the hepatic LPS load. Therefore, liver occlusion experiment was designed to increase the hepatic LPS load per functional liver mass. After iv application of the standard non-lethal LPS dose (2 mg/kg) to the rats, the LPS accumulation per liver mass was increased by either resection of 70% liver mass to increase the LPS load/ liver mass, and by occluding selected liver lobes to prevent the LPS uptake (Additional file 1: Figure S1).

**Fig. 2:**
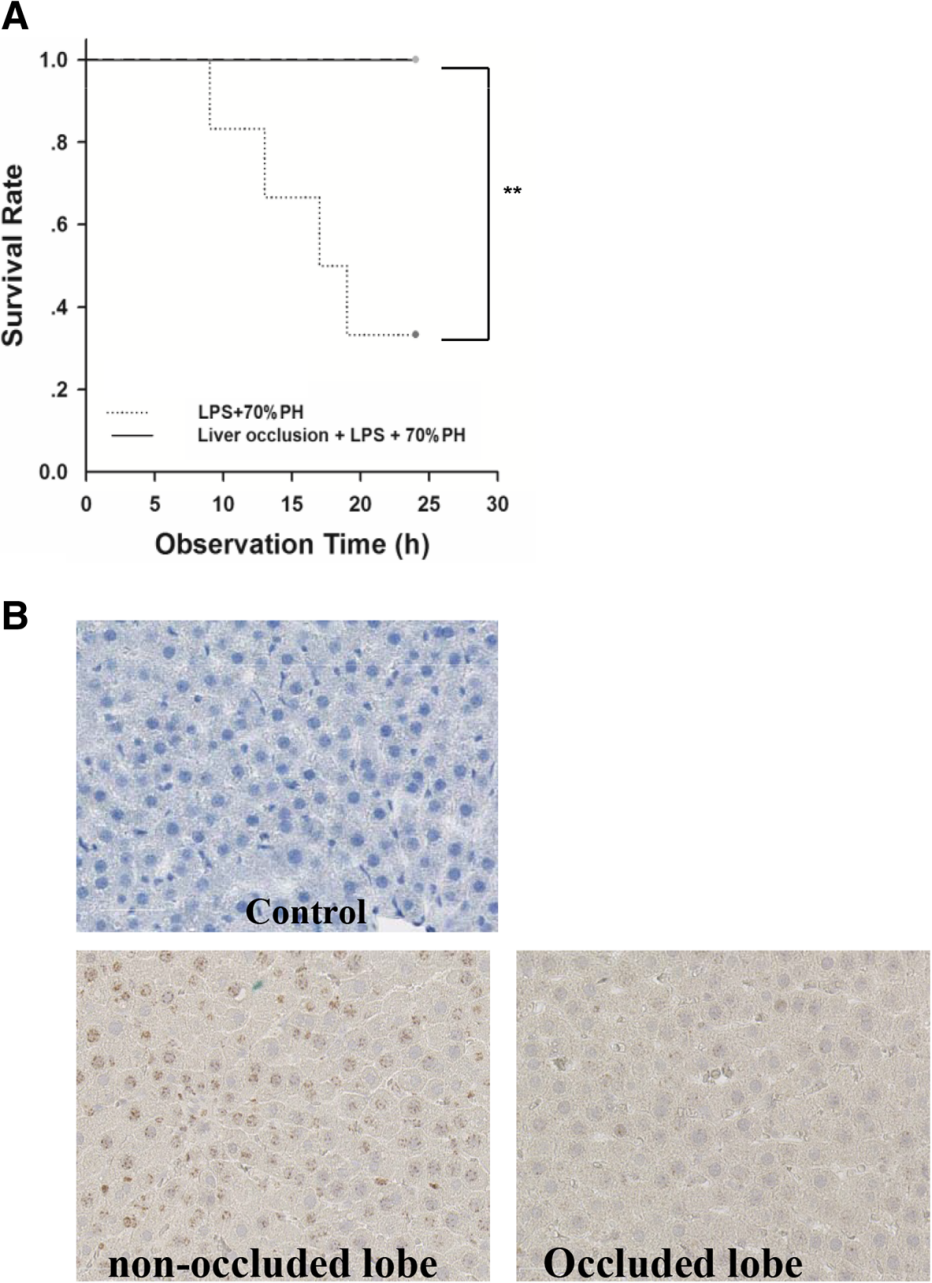
LPS induced mortality was determined by the hepatic LPS uptake. **a** Survival rate of rats in LPS + 70%PH group, and liver occlusion+ LPS + 70%PH group. Decrease of hepatic LPS uptake by liver occlusion reversed the mortality induced by 2 mg/kg LPS after 70%PH; **b** LPS IHC was performed to indicate the hepatic LPS uptake in occluded and un-occluded liver lobes. Liver occlusion by clamping right lobes for 30 min decreases the hepatic LPS uptake (*n* = 6 per group). **P<0.01

We observed that increasing the LPS load per liver mass by performing 70% PH caused the death of 70% of the rats within 24 h. However, preventing hepatic LPS uptake by occluding liver lobes reduced the systemic injury, and increased the survival rate to 100% (Fig. 2a). LPS uptake as indicated by LPS-IHC was significantly lower in the occluded liver lobes (less positive staining) than in the non-occluded liver lobes (moderate positive staining) 1 h after LPS injection (Fig. 2b).

**Fig. 3:**
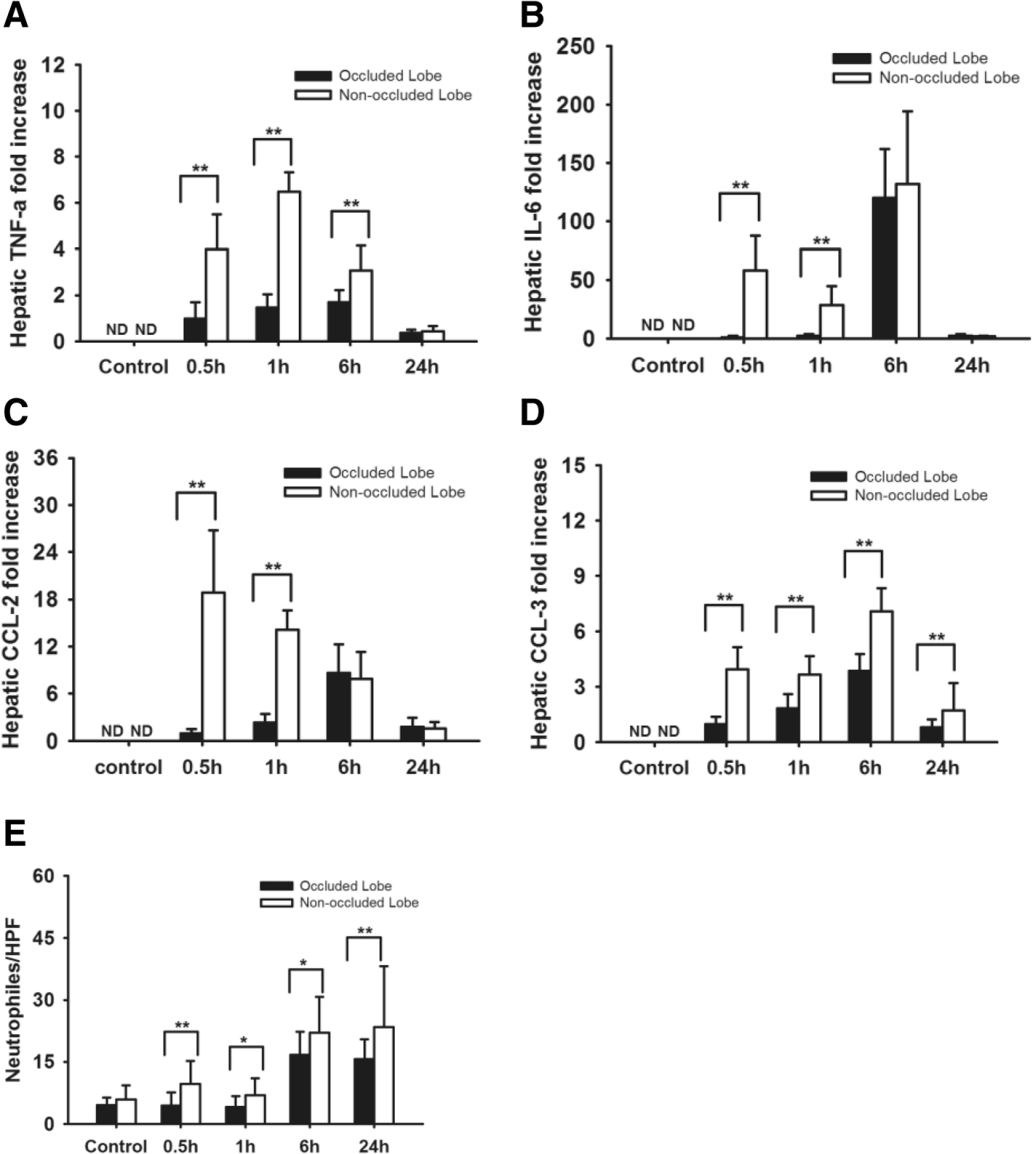
The systemic inflammatory response and organ damage was decreased by reducing LPS-load in the liver. **a**-**b** liver occlusion inhibited the LPS induced hepatic TNF-a (**a**), and IL-6 (**b**) mRNA expression. **c**-**d** The expression of CCL-2 and CCL-3 mRNA levels in liver tissues. LPS induced upregulation of hepatic CCL-2 (**c**) and CCL-3 (**d**) mRNA expression was inhibited by liver occlusion. **e** The hepatic influx of neutrophil was analyzed by counting ASDCL positive cells. The neutrophil infiltration to occluded liver lobes was significantly lower compared to non-occluded lobes. *P<0.05, **P<0.01

A kinetic experiment was performed to investigate the local inflammatory response. In the kinetic experiment, the local expression of pro-inflammatory cytokines was compared between the occluded liver lobes and non-occluded liver lobes 0.5 h, 1 h, 6 h, and 24 h after the re-perfusion. The results revealed that the expression of TNF-α, IL-6, CCL-2, and CCL-3 mRNA was significantly higher in non-occluded lobes compared to the occluded lobes (Fig. 3a-d). Moreover, the infiltration of neutrophils as visualized by ASDCL staining in non-occluded liver lobes was also significantly higher than in the occluded liver lobes (Fig. 3e).

### The systemic inflammatory response and organ damage was decreased by reducing the LPS-load in the liver

**Fig. 4:**
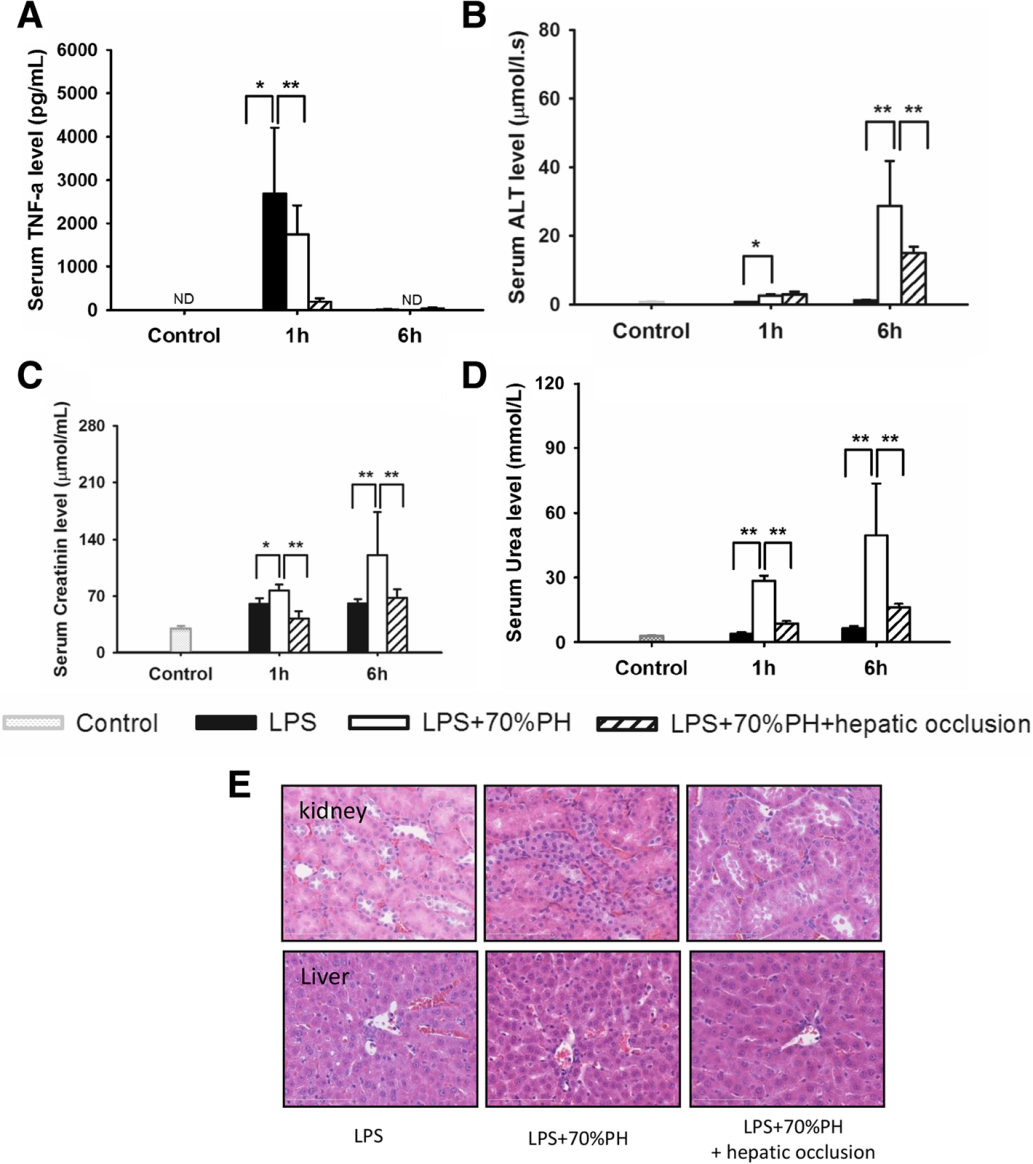
Blockade of hepatic LPS uptake by liver occlusion decreased systemic inflammatory response. **a** Blockade of hepatic LPS uptake by liver occlusion decreased the serum TNF-a levels. **b** Blockade of hepatic LPS uptake by liver occlusion decreased the release of liver enzyme ALT. **c**-**d** blockade of hepatic LPS uptake by liver occlusion decreased the creatinine (**c**), and urea (**d**) levels. **e** The morphological changes was evaluated by performing HE staining in kidney, and liver. Representative images from 6 rats per group were selected. Original magnification × 200. *p < 0.05, **p < 0.01 Data are shown as mean ± SD

Using samples from the same experiment, the systemic inflammatory response, and tissue injury was compared. As shown in Fig. 4a, the serum TNF-α levels in LPS + 70%PH + liver occlusion group were significantly lower than in LPS only group, and LPS + 70%PH group. Occlusion of RLs decreased the liver and kidney damage, as indicated by decreased serum ALT, Creatinine, and urea acid levels (Fig. 4b-d) The organ damage was also evaluated by the morphological changes in liver, and kidney (Fig. 4e), respectively. Rat subjected to LPS injection followed by 70% PH showed severe organ damage after 6 h observation time in liver, and kidney, such as marked vacuolar degeneration of tubular cells in kidney, sinusoidal dilatation and erythrocyte congestion in liver. Blockade of LPS hepatic uptake by liver occlusion was associated with substantially less damage as indicated by the minimal histopathological changes.

## Discussion

In the present study, we developed a surgical model for investigating the role of liver in initiating LPS induced systemic injury. We increased the hepatic LPS exposure either by resection or by occlusion of selected liver lobes.

### In the first step, we performed 30, 70, and 90% liver resection

Reduction of liver mass induced an LPS-response of increasing severity leading to decreased serum TNF-α, and the death of rats. Reduction of the liver mass by 30% caused 100% survival rate of the rats. Further liver mass reduction by 70% PH did not only decrease serum TNF-α, but also reduced the survival rate to 33%. Extended resection of 90% of the liver mass caused the death of all animals, in other words a survival rate of 0%. In this case, the serum TNF-α was undetectable.

### Systemic TNF-α levels were inversely related to the remnant liver mass, but not to the severity of inflammatory injury

TNF-α is the one of most important inflammatory cytokines, and is frequently used as indicator of the inflammatory response. In animal models, administration of LPS induces the expression and release of TNF-α [7]. Most authors observed the peak of serum TNF-α level as early as 0.5–2 h after LPS injection [8, 4]. In contrast, we noticed in our study that the serum TNF-α level after LPS injection and liver resection did not reflect the severity of the inflammatory injury, and survival rate. Interestingly, the serum TNF-α level was inversely related to the extent of PH. Previous studies have shown that decreasing the liver blood flow by portacaval shunt down regulate the systemic TNF-α levels [9]. The portal vein ligation decreased LPS induced TNF-α in rat model [10]. These observations suggested that the severity of the LPS-response in terms of organ damage was determined by the remnant liver mass and was not related to the elevation of TNF-α level. Therefore we must conclude that elevation of TNF-α is apparently not essential for the induction of inflammatory organ injury.

### In the next step, we established a new surgical model allowing for selective hepatic uptake of LPS

The model consisted of transient blockade of the right portal vein followed by the LPS injection and liver resection. As mentioned before, increasing the hepatic LPS exposure by reducing the liver mass by 70% PH was associated with an increased inflammatory response and severe tissue injury in kidney. Decreasing hepatic LPS exposure of the remnant right liver lobes by clamping the right portal vein attenuated the local and systemic inflammatory response, and increased the survival rate. Subsequently, the LPS uptake in un-occluded liver lobes was higher than in the occluded liver lobes. These observations suggested that the LPS-exposure of the small remnant liver might play a decisive role in the initiation and severity of the LPS induced inflammatory injury. LPS induces a systemic inflammatory response, which causes multi-organ damage, and ultimately multi-organ dysfunction [11]. However, it is unknown that whether the organ dysfunction and failure is interdependent. In our experiments, inhibition of hepatic LPS uptake by right lobe occlusion significantly decreased the systemic inflammatory response, and the tissue damage in kidney, which was associated with a significantly increased survival rate. Studies demonstrated that the impaired liver function might responsible for the damage of other organs. Hepatic STAT-3 and RelA knock out increased the mortality in a mouse pneumonia model [12]. Hepatic deficiency of atg5 significantly reduced the survival rate in a mouse CLP model [13]. Yan et al. indicated, that liver dysfunction after sepsis is an independent risk factor for multiple organ dysfunction and sepsis-induced death [14].

Therefore, our newly created surgical model to investigate LPS-induced inflammation and systemic organ injury will be helpful for better understanding the development of multi-organ dysfunction. Although the inflammatory response and subsequent systemic organ damage is an important process in SIRS and sepsis, establishment of novel surgical animal models for better investigation their interdependency was not really pursued. The traditional animal models focus on the effect of pathogens in the whole organism level [11], without the possibility to interfere with the function of single organs. The use of genetically modified animals can only reflect the role of single or few target genes in the complex inflammatory condition. Our newly established surgical model allows investigating the role of liver in the initiation of systemic inflammatory response. Using this model will facilitate the research in understanding the pathophysiology of sepsis, and improving the therapy. By using this model, more complex experiments could be designed and performed in order to investigate the function of liver in the development of SIRS and severe sepsis.

Our results support, that the LPS induced systemic inflammatory injury was determined by hepatic LPS response. The lethal effect of LPS was reversed by reducing the LPS load in the liver. These observations may be useful for planning future experimental studies and clinical trials in which the role of novel therapeutic agents or protocols are being evaluated.

## Additional file

Additional file 1: **Figure S1**. Experimental design to investigate the effect of liver occlusion on hepatic LPS uptake. (TIF 138 kb)

## Abbreviations

ASDCL: Naphthol-AS-D-chloroacetate
AST: Alanine transaminase
ELISA: Enzyme-linked Immunosorbent Assay
HPF: High-power fields
HPRT: Hypoxanthine guanine phosphoribosyltransferase
IHC: Immunohistochemistry
LBP: LPS binding protein
LEW: Lewis
LPS: Lipopolysaccharide
NF-κB: Nuclear factor kappa-light-chain-enhancer of activated B cells
PCR: Quantitative polymerase chain reaction
SIRS: Systemic inflammatory response syndrome
TLR: Toll like receptor
