# Supplementary figures and images for: The severity of LPS induced inflammatory injury is negatively associated with the functional liver mass after LPS injection in rat model
Source: J Inflamm (Lond). 2018 Nov 15;15:21. doi: 10.1186/s12950-018-0197-4 (PMC6238277; doi:10.1186/s12950-018-0197-4)

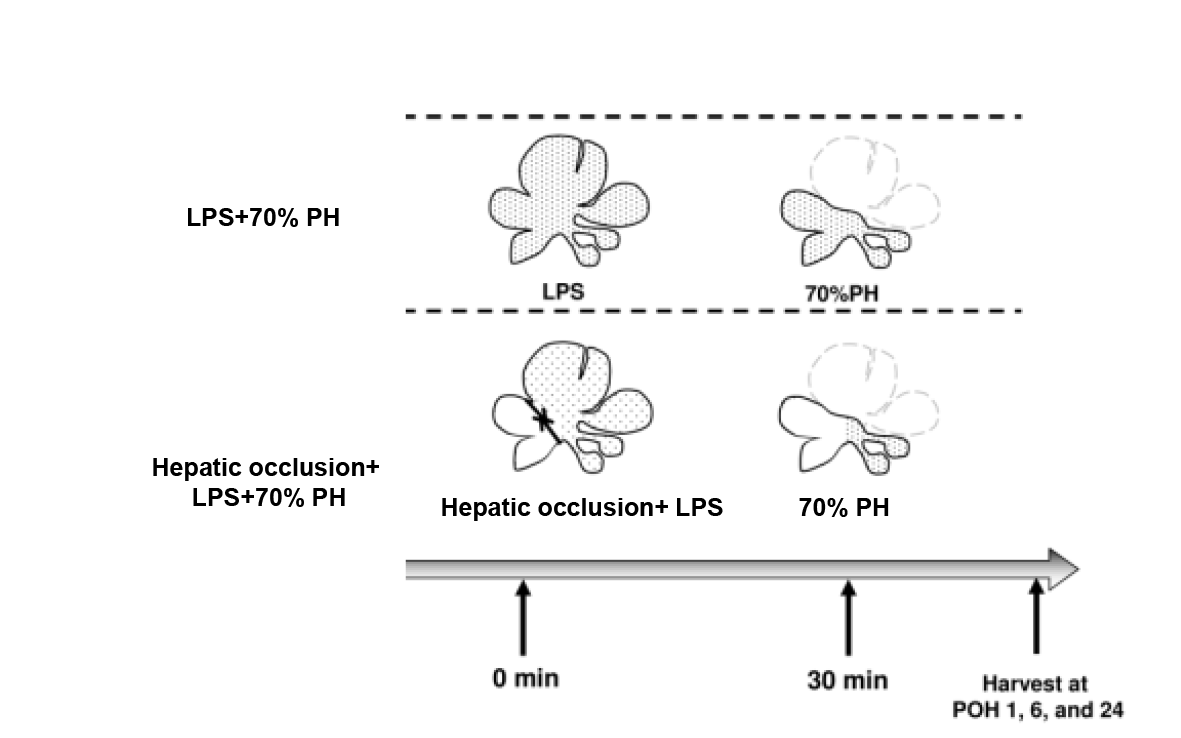

Supplement: Supplementary file 1 — Figure S1. Experimental design to investigate the effect of liver occlusion on hepatic LPS uptake. (TIF 138 kb) [file 12950_2018_197_MOESM1_ESM.tif]
